# Fast construction of voxel-level functional connectivity graphs

**DOI:** 10.1186/1471-2202-15-78

**Published:** 2014-06-19

**Authors:** Kristian Loewe, Marcus Grueschow, Christian M Stoppel, Rudolf Kruse, Christian Borgelt

**Affiliations:** 1 Department of Neurology, Experimental Neurology, Otto-von-Guericke Universität, Leipziger Str. 44, 39120 Magdeburg, Germany; 2 Department of Knowledge and Language Processing, Otto-von-Guericke Universität, Magdeburg, Germany; 3 Department of Economics, University of Zürich, Zürich, Switzerland; 4 Department of Psychiatry and Psychotherapy, Charité - Universitätsmedizin, Berlin, Germany; 5 European Centre for Soft Computing, Mieres (Asturias), Spain

**Keywords:** Functional connectivity, Graph theory, Tetrachoric correlation, Resting-state, fMRI

## Abstract

**Background:**

Graph-based analysis of fMRI data has recently emerged as a promising approach to study brain networks. Based on the assessment of synchronous fMRI activity at separate brain sites, functional connectivity graphs are constructed and analyzed using graph-theoretical concepts. Most previous studies investigated region-level graphs, which are computationally inexpensive, but bring along the problem of choosing sensible regions and involve blurring of more detailed information. In contrast, voxel-level graphs provide the finest granularity attainable from the data, enabling analyses at superior spatial resolution. They are, however, associated with considerable computational demands, which can render high-resolution analyses infeasible. In response, many existing studies investigating functional connectivity at the voxel-level reduced the computational burden by sacrificing spatial resolution.

**Methods:**

Here, a novel, time-efficient method for graph construction is presented that retains the original spatial resolution. Performance gains are instead achieved through data reduction in the temporal domain based on dichotomization of voxel time series combined with tetrachoric correlation estimation and efficient implementation.

**Results:**

By comparison with graph construction based on Pearson’s *r*, the technique used by the majority of previous studies, we find that the novel approach produces highly similar results an order of magnitude faster.

**Conclusions:**

Its demonstrated performance makes the proposed approach a sensible and efficient alternative to customary practice. An open source software package containing the created programs is freely available for download.

## Background

The functioning of the human brain relies on the interplay and integration of numerous individual units in a complex network. Insights into its topology are thus essential to promote our understanding of the brain in general, as well as its maladaptive states associated with dysfunction and disease. An increasingly popular approach to the analysis of functional brain networks is based on the framework of graph theory [1-4]. A graph is a mathematical structure designed for modelling pairwise relationships, known as edges, between an assortment of objects, referred to as nodes. In applications to fMRI, the node set is defined as a collection of brain sites, and edges are established by measuring internodal functional connectivity [5] based on the regions’ associated time series. The obtained functional connectivity graph, serving as a simple model of the brain’s functional organization in a complex network, is subsequently examined drawing on a rich collection of graph-theoretical metrics that target various aspects of its topology [6]. Several studies indicate, for instance, that the brain’s functional network conforms to a small-world architecture [1,2,7,8]. Beyond that, the usefulness of graph-based functional connectivity analyses has been demonstrated in applications to brain development and aging [9-11], gender differences [12], intellectual performance [13], and neurological disorders, such as Alzheimer’s disease [14,15] or schizophrenia [16,17].

In most previous studies, functional connectivity graphs have been constructed at the level of regions [1,7,10,14,18], meaning that graphical nodes are defined based on a parcellation of the brain into regions of interest (ROI), each consisting of several voxels. Due to the limited number of nodes, such analyses are computationally inexpensive and their results are comparatively easy to visualize and interpret. However, region-level nodes involve mixing fMRI time series from the incorporated voxels, thus obliterating more detailed spatial information [4,19]. ROI-based analyses are therefore highly dependent on the quality of the parcellation: If ROI boundaries and actual functional boundaries are inconsistent, the results can be erroneous [20]. Voxel-level analyses, in contrast, are not subject to these limitations, since the parcellation inherent to the original data is used for node definition [8,11,13,15,21-23]. Consequently, voxel-level graphs provide a finer model of the brain’s functional network organization, since the original spatial resolution of the fMRI data is preserved [4,24].

Because of the large number of nodes, the construction and analysis of voxel-level graphs can involve considerable computational efforts. In response, the computational burden has often been reduced by sacrificing spatial resolution (using relatively large voxels to begin with or reslicing the data to a lower resolution) thus reducing the number of nodes in the graph [15,25-27]. While reduction of spatial resolution is undesirable in general (given that the main advantage of fMRI as compared to other methods such as EEG/MEG is its superior spatial resolution), it can even render a study infeasible, e.g., when investigating very small brain structures or different regions that lie in close proximity to each other. Efficient algorithms and implementations are therefore required in order to take full advantage of the data’s original spatial resolution [24].

Here, we propose a novel approach aiming to increase the computational efficiency of voxel-level graph construction by combining time series dichotomization, tetrachoric correlation estimation, and efficient implementation, while retaining the full spatial resolution of the data. Comparison with conventional graph construction (as carried out in previous studies) shows that the new approach not only produces highly similar results, but also executes an order of magnitude faster.

## Methods

This section consists of three parts. We begin with a short introduction to voxel-level functional connectivity graphs and explain their construction from fMRI data. In particular, it is established how time series dichotomization can be combined with tetrachoric correlation estimation to efficiently measure functional connectivity. The second part describes analyses comparing Pearson’s *r* and the tetrachoric correlation coefficient *r*_*t*_ (1) as correlation estimators in the controllable environment of synthetic data, (2) as measures of functional connectivity in the context of graph construction from fMRI data, and (3) with respect to the similarity of graphs resulting from (2). The last part provides implementation details regarding the programs created for this study and assesses their computational performance.

### Voxel-level graph construction

Formally, an undirected binary graph is defined as an ordered pair *G*_*B*_=(*N*,*E*), comprised of a set of nodes *N* and a set of pairwise internodal connections, or edges, *E*. Individual edges are unordered pairs {*i*,*j*}, where *i*,*j*∈*N*. *G*_*B*_ can be represented by a binary adjacency matrix **B**^|*N*|×|*N*|^=(*b*_*i*,*j*_), where *b*_*i*,*j*_∈{0,1}, *i*,*j*∈*N*, and *b*_*i*,*j*_=1 indicates that {*i*,*j*}∈*E*, i.e., that a connection between the two nodes *i* and *j* exists. In order to represent not only the presence or absence of connections but also their strength, a graph can be extended by assigning a weight to each edge.

**Figure 1:**
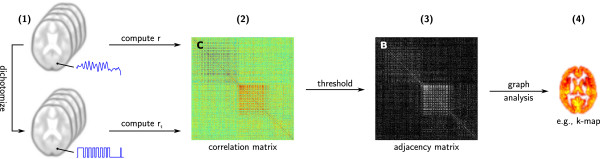
**Construction and analysis of voxel-level functional connectivity graphs.** Starting with the preprocessed fMRI data, all gray matter voxels are defined as graphical nodes **(1)**. Using their associated time series, pairwise internodal functional connectivity is measured in terms of linear correlation. Typically, this is done using Pearson’s *r*. Alternatively, one can derive binary time series via median-based dichotomization and employ tetrachoric correlation estimation (*r*_*t*_). In both cases, the result is a correlation matrix **(2)** representing the pairwise functional connectivity between nodes. A binary undirected graph, represented by a binary adjacency matrix **(3)**, is obtained via thresholding. Based on the adjacency matrix, graph-theoretical metrics, such as the node degree *k*, are computed **(4)**.

In applications to fMRI data, graph-based analyses rely on the derivation of a graphical representation of the brain’s functional network, which is then examined in terms of graph theory (Figure 1). Voxel-level functional connectivity graphs are constructed based on individual voxels as nodes, that is, the set of nodes *N* is a collection of voxels. In the literature, *N* is often defined as all in-brain voxels, or all gray matter (GM) voxels. Functional connectivity is estimated between all pairs of nodes based on their corresponding time series using a measure of association.

To construct a binary graph, edges are established by thresholding the functional connectivity estimates: Two nodes are connected by an edge if their functional connectivity exceeds a given threshold. Alternatively, one can take the pairwise functional connectivity matrix as a basis for a weighted graph, thus conserving the strength of individual functional connections between nodes [28]. For simplicity, and in a manner consistent with the majority of previous work on voxel-level functional connectivity graphs, we presently focus on binary graphs for our analysis.

#### Measuring functional connectivity

In most previous studies investigating voxel-level functional connectivity graphs, internodal functional connectivity is measured using Pearson’s sample correlation coefficient *r*[2,8,13,15,21-27,29-31]. When using Pearson correlation as a measure of functional connectivity, it seems sensible to assume bivariate normality with respect to the distribution of pairwise observations arising from each pair of voxel time series. This is because Pearson correlation may be a poor measure of association if the data are not normally distributed [32]. Encouragingly, in a recent study employing region-level graphs, data for the most part appeared to meet the assumption of bivariate normality [33].

Assuming bivariate normality between pairs of voxels, an alternative correlation estimator, the tetrachoric correlation coefficient *r*_*t*_[34], can be used instead of *r*. Given two dichotomous variables *x*_*d*_ and *y*_*d*_, *r*_*t*_ estimates the correlation of the latent continuous-valued variables *x*_*c*_ and *y*_*c*_ associated with *x*_*d*_ and *y*_*d*_, under the assumption that *x*_*c*_ and *y*_*c*_ follow a bivariate normal distribution. Thus, if we dichotomize, i.e., binarize, the voxel time series data, *r*_*t*_ can be used to estimate the pairwise correlation of the original continuous-valued time series from the dichotomized ones.

Consider two voxels, *v* and *w*, and their corresponding time series, ***s***_*v*_ and ***s***_*w*_. Using the medians of ***s***_*v*_ and ***s***_*w*_, i.e., ${\tilde{s}}_{v}$ and ${\tilde{s}}_{w}$, as dichotomization thresholds, we obtain the binary time series ***d***_*v*_ and ***d***_*w*_. Formally, *d*_*v*,*k*_=1 if the signal intensity value *s*_*v*,*k*_ amounts at least to ${\tilde{s}}_{v}$ and *d*_*v*,*k*_=0 otherwise, where *k*∈{1,…,*T*} and *T* is the number of acquired fMRI volumes [35]. See Section S.1 for details.

**Figure 2:**
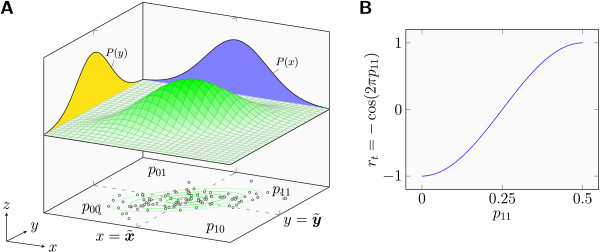
**Dichotomization and tetrachoric correlation estimation.** Consider a sample ((*x*_1_,*y*_1_), (*x*_2_,*y*_2_),…,(*x*_*T*_,*y*_*T*_)) of size *T* where (*x*,*y*) is distributed according to bivariate normality. Further, let ***x***=*x*_1_,*x*_2_,…,*x*_*T*_ and ***y***=*y*_1_,*y*_2_,…,*y*_*T*_ denote the samples from *x* and *y*, respectively. Using $\tilde{x}$ and $\tilde{y}$, as thresholds, ***x*** and ***y*** can be dichotomized resulting in the binary samples ***x***_*d*_ and ***y***_*d*_. A: As an example, the density of a bivariate normal distribution (*ρ*=0.7) is shown (top, 3D curve) along with a sample (bottom, points in the *x*-*y*-plane) drawn from that distribution. By virtue of the two lines $x=\tilde{x}$ and $y=\tilde{y}$, the *x*-*y*-plane is divided into four quadrants, such that the counts of sample points per quadrant form a 2×2 contingency table. The (relative) frequencies in the contingency table can also be expressed in terms of ***x***_*d*_ and ***y***_*d*_ (e.g., *n*_11_ is the number of indices where both ***x***_*d*_ and ***y***_*d*_ are equal to 1, and *p*_11_=*n*_11_*T*^−1^). The probability masses corresponding to the table’s relative frequencies are equal to the respective partial volumes belonging to the four quadrants in the *x*-*y*-plane under the bivariate normal’s curve. The tetrachoric correlation coefficient *r*_*t*_, for which these partial volumes resemble the relative frequencies in a given table, is an estimate of the population correlation parameter *ρ* belonging to the underlying distribution. B: Relationship between *p*_11_ and *r*_*t*_. Given ***x***_*d*_ and ***y***_*d*_, *r*_*t*_ can be found using *r*_*t*_=− cos(2*πp*_11_). For details see text.

By virtue of ${\tilde{s}}_{v}$ and ${\tilde{s}}_{w}$, the pairs (*s*_*v*,*k*_,*s*_*w*,*k*_) are divided into four partitions corresponding to four quadrants in the *x*-*y*-plane of a Cartesian coordinate system (Figure 2A, bottom). Thus, by counting the number of points falling into each quadrant, a pair of voxels gives rise to a 2×2 contingency table, the (relative) frequencies of which can be expressed in terms of ***d***_*v*_ and ***d***_*w*_. For example, *n*_11_, the frequency of points in time where *s*_*v*,*k*_ and *s*_*w*,*k*_ amount at least to ${\tilde{s}}_{v}$ and ${\tilde{s}}_{w}$, respectively, is given by $n_{11}=\overset{T}{\underset{k=1}{\sum }}d_{v,k}\cdot d_{w,k}$. In other words, *n*_11_ is the number of points where both ***d***_*v*_ and ***d***_*w*_ are equal to 1, yielding the associated relative frequency *p*_11_ through $p_{11}=\frac{n_{11}}{T}$.

The probability masses corresponding to the table’s relative frequencies are equal to the respective partial volumes belonging to the four quadrants in the *x*-*y*-plane under the curve representing the bivariate normal distribution (Figure 2A). The correlation coefficient *r*_*t*_, for which these partial volumes resemble the relative frequencies in a given table, is an estimate of the population correlation *ρ* belonging to the underlying distribution. Since a 2×2 contingency table is uniquely defined by the marginal probabilities and one joint probability, *r*_*t*_ can be found by solving, e.g., $p_{11}=\int_{\Phi^{-1}(p_{∙0})}^{\infty }\int_{\Phi^{-1}(p_{0∙})}^{\infty }f(z_{x},z_{y})dz_{x}dz_{y}$, where *Φ* is the standard normal distribution function, *Φ*^−1^ is its inverse, and *f* is the probability density function of the bivariate normal distribution. While this would typically be solved using numerical techniques, an analytical solution, *r*_*t*_=− cos(2*πp*_11_), exists for the case under consideration (Figure 2B). See Section S.2 for details.

Given a pair of voxels, we can determine *p*_11_ from the dichotomized time series and use the relationship *r*_*t*_=− cos(2*πp*_11_) in order to obtain *r*_*t*_. As a consequence, *r*_*t*_ can be used instead of *r* to estimate pairwise functional connectivity in the process of graph construction from fMRI data (Figure 1).

### Simulations

Building upon the theoretical considerations presented above, we analyzed the characteristics of *r*_*t*_ in the controllable environment of synthetic data. More specifically, we assessed its usefulness relative to *r* in estimating the correlation parameter *ρ* of bivariate normal populations of known properties.

#### Data

Bivariate normal populations were generated, such that each of them exhibited a predefined population correlation *ρ*, where *ρ*∈{−0.99,−0.98,…, 0,…,0.98,0.99}. Then, 10000 bivariate samples of size *T* (one sample represents a pair of time series of length *T*) were randomly drawn from each of the populations. For each sample, *r* and *r*_*t*_ were calculated. Prior to calculating the latter, the data were dichotomized as described above. The entire procedure was conducted separately for two different sample sizes, *T*=100 and *T*=300, resulting in two data sets, where the choice of these numbers was guided by the parameters of the real fMRI data we analyzed.

#### Correlation estimation

For each data set and estimator $\hat{\rho }\in \{r,r_{t}\}$, joint histograms ($\hat{\rho },\rho$) with associated marginal histograms were calculated. For the joint histograms a linearly spaced 199×199 grid was used, such that bin centers in both dimensions corresponded to correlations exhibited by the generated bivariate populations. For each estimator, means and standard deviations, as well as mean signed differences $\text{MSD}(\hat{\rho },\rho )$ were calculated per *ρ*-bin. Mean signed differences are defined as $\text{MSD}(\hat{\rho },\rho )=n^{-1}\overset{n}{\underset{i}{\sum }}({\hat{\rho }}_{i}-\rho )$, where *n* is the number of samples per *ρ*-bin, i.e., *n*=10000, and ${\hat{\rho }}_{i}$ is the correlation estimate for sample *i*. Since *ρ* is not known in the case of real data, additional joint histograms (*r*_*t*_,*r*) were calculated in order to facilitate comparability with respect to real data applications. As both estimators exhibit errors with respect to *ρ*, Deming regression^a^ was used in order to fit a linear relationship to the (*r*_*t*_,*r*) data.

### Application to fMRI data

Comparative graph-based analyses of resting-state fMRI data were carried out based on *r* vs. *r*_*t*_ as measures of functional connectivity.

#### Data

MRI data were obtained from the “1000 Functional Connectomes Project” repository [36,37]: We used the “Cambridge” and “Pittsburgh” data sets, contributed by R.L. Buckner and G. Siegle, respectively. These data sets contain resting-state fMRI data from 198 subjects (75 males/123 females, ages: 18–30 years; imaging parameters: TR = 3s, voxel size =3×3×3 mm^3^, number of slices = 47; number of volumes =119) and 17 subjects (10 males/7 females, ages: 25–54; imaging parameters: TR = 1.5s, voxel size =3.125×3.125×3.2 mm^3^, number of slices =29; number of volumes =275), respectively. Both data sets also include anatomical scans for each subject.

#### Preprocessing

Using SPM8 [38] functional data were motion-corrected by alignment to the mean functional image, then anatomical scans were coregistered to the mean functional image and segmented. In order to account for low frequency intensity drifts and high frequency noise, frequencies below 0.01Hz and above 0.1Hz were removed from the voxels time series by band-pass filtering, as is customary for resting-state data [39]. In order to minimize the impact of preprocessing on the data’s correlation structure, we refrained from spatial smoothing and spatial normalization [8,27].

#### Correlation estimation

Based on *r* and *r*_*t*_ as a measure of functional connectivity, two voxel-level graphs were constructed for each subject from the two data sets. Nodes were defined as supra-threshold voxels in the subject-specific GM probability maps obtained from the segmentation (threshold *θ*_*G* *M*_=0.2). To measure internodal functional connectivity, two correlation matrices, **C**_*r*_ and $C_{r_{t}}$, were calculated based on all pairwise correlations between nodes. **C**_*r*_ was obtained by calculating Pearson correlations based on the voxels’ associated continuous-valued time series from the preprocessed fMRI data, and $C_{r_{t}}$ was obtained analogously, except that tetrachoric correlations were calculated instead of Pearson correlations, and binary voxel time series were used instead of continuous-valued ones. Again, binary voxel time series were derived from the continuous-valued ones through median-based dichotomization. In order to compare **C**_*r*_ and $C_{r_{t}}$, their entries were used to calculate joint histograms (*r*_*t*_,*r*) in the same fashion as for the synthetic data.

#### Functional connectivity graphs

Subject-specific binary functional connectivity graphs, **B**_*r*_ and $B_{r_{t}}$, were derived from **C**_*r*_ and $C_{r_{t}}$, respectively, via density-based thresholding: The density *κ* of a binary undirected graph **B** is the proportion of potential edges that actually exist, i.e., $\kappa =\frac{2\cdot |E|}{\left|N|\cdot (|N|-1\right)}$. In order to facilitate comparability across graphs, an individual correlation threshold *θ* was determined for each correlation matrix, such that the resulting binary graphs exhibited the same density *κ*. Given **C**, where $C\in \{C_{r},C_{r_{t}}\}$, and *θ*, the entries of **B** are given by *b*_*i*,*j*_=1, if *c*_*i*,*j*_>*θ*, and *b*_*i*,*j*_=0 otherwise, where 1≤*i*,*j*≤|*N*|.

#### Node degree maps

In graph-based fMRI functional connectivity analyses, one of the most popular graph-theoretical metrics is the node degree, or degree centrality, a measure aiming to characterize the importance of individual nodes in a binary graph. Given a binary graph **B**, the degree *k*_*i*_ of a node *i* is defined as the number of nodes that are connected to *i* via an edge, or, more formally, $k_{i}=\overset{\left|N\right|}{\underset{j}{\sum }}b_{i,j}$, where *i*,*j*∈*N* and *i*≠*j*[40]. The node degree has recently been employed in several neuroimaging studies aiming to identify potential hub regions in the human brain [27,31].

Here, node degrees *k* were calculated for all subject-specific functional connectivity graphs **B**_*r*_ and $B_{r_{t}}$. Degrees were standardized in order to afford comparable scaling across subjects [15]. The spatial distribution of degrees was analyzed by constructing *k*-maps in individual brain space for each subject. In order to generate group average *k*-maps for each data set (Cambridge and Pittsburgh), the subject-specific *k*-maps were spatially normalized to ICBM^b^ -template space based on transformation parameters estimated with respect to the mean functional image using SPM8 [41-43]. Since the normalized *k*-maps have different overlaps due to anatomical differences and differing GM masks, a varying number of subjects "supports" each standard space voxel. Thus, group-level *k*-maps were derived by voxel-wise averaging of the *k*-values from the supporting subjects. For enhanced reliability, *k*-values of voxels supported by less than 20% of all subjects were discarded.

### Implementations

The most time-consuming step when constructing a graph from fMRI data consists in the computation of a functional connectivity matrix, which here corresponds to the computation of a correlation matrix based on *r* or *r*_*t*_. In the following, the programs created for calculating the voxel-level pairwise correlation matrices **C**_*r*_ and $C_{r_{t}}$ will be referred to by pcc and tetracc, respectively. For both programs we opted to store only the upper triangular part of **C**, in order to save memory. In doing so, no information is lost, since **C** is symmetric. Because efficient implementation plays an important role when aiming to accelerate large-scale analyses, implementation was conducted using the C programming language, providing Matlab integration via its C interface MEX. A Matlab toolbox and C sources are available for download [44].

#### Calculation of *C*_*r*_

Pearson’s sample correlation coefficient *r* is calculated for a pair of voxels *v* and *w* using

$$r(s_{v},s_{w})=\frac{\overset{T}{\underset{k=1}{\sum }}(s_{v,k}-{\bar{s}}_{v})(s_{w,k}-{\bar{s}}_{w})}{\sqrt{\overset{T}{\underset{k=1}{\sum }}{\left(s_{v,k}-{\bar{s}}_{v}\right)}^{2}}\cdot \sqrt{\overset{T}{\underset{k=1}{\sum }}{\left(s_{w,k}-{\bar{s}}_{w}\right)}^{2}}}\;.$$

To avoid redundant operations, subexpressions depending on one voxel only are precalculated for all voxels before computing **C**_*r*_.

In order to take advantage of the processor’s cache without the need for explicit knowledge about its size, we adopted a so-called *cache-oblivious algorithm*[45,46] to compute the correlation matrix, rather than explicit *blocking* (with a predetermined block size that optimally fits the cache). The core idea is to recursively divide the problem so that the computations are carried out on smaller and smaller blocks of data. Given that the minimum block size is small enough, there is a division step from which on all computations use only data that fits into the processor cache (regardless of its size), thus making optimal use of the cache by localizing the computations. The division scheme we implemented is illustrated in Additional file 1: Figure S1, which shows the first three steps of dividing the upper triangle of the correlation matrix.

In addition, we exploited SSE2 (Streaming SIMD Extensions version 2, where SIMD stands for Single Instruction Multiple Data) and AVX (Advanced Vector eXtensions) instructions (on processors that support them^c^), which allow for parallelization on a single core by carrying out the same operation on multiple data elements in parallel (also known as vectorized computations). Using SSE2 (AVX), the computation of the numerator of the correlation coefficient can be parallelized by computing four (eight) sums in parallel (if the float data type is used; for double, two and four sums, respectively, can be computed in parallel). The procedure is illustrated in Additional file 1: Figure S2 for SSE2 using float or AVX using double (four sums in parallel).

#### Calculation of $C_{r_{t}}$

For each pair of voxels, *r*_*t*_ is computed in three steps. First, the bitwise AND operator is applied to the voxels’ associated binary time series. Second, the set bits in the result are counted to obtain *n*_11_. Third, *r*_*t*_ is retrieved from a lookup table of the function *r*_*t*_=− cos(2*πn*_11_*T*^−1^). The table is indexed by *n*_11_ and contains the corresponding *r*_*t*_ values for those values that *n*_11_ can attain. Depending on *T* being even or odd, these are $0,1,2,\ldots ,\frac{T}{2}$ or $1,2,3,\ldots ,\frac{T+1}{2}$, respectively.

Storing the binary time series in integers of, e.g., 32 bit, 32 points in time can be processed in parallel, so that the above three steps need to be executed only ⌈*T*/32⌉ times per pair. Hence, it seems conceivable that the computational cost in terms of CPU time could be lower for the calculation of $C_{r_{t}}$ than for the calculation of **C**_*r*_.

Following the procedure outlined above, two programs, tetracc/32 and tetracc/128 were created. tetracc/32 uses 32 bit integers for storing binary time series and a 16 bit lookup table for bit-counting. tetracc/128 uses __mm128i variables (holding 128 bit each) for time series storage. Using the intrinsics _mm_and_si128 and _mm_popcnt_u64 for bitwise AND and bit-counting, respectively, it is expected to improve over tetracc/32, since more data can be processed in parallel and no extra memory access (lookup table) is needed. While tetracc/32 is platform-independent, tetracc/128 is only applicable on fairly modern CPUs, because _mm_and_si128 and _mm_popcnt_u64 depend on the availability of SSE2 and POPCNT instructions, respectively^d^.

#### Parallel versions

For additional performance gains, parallel versions of pcc and tetracc have been implemented using multiple threads. This aspect is, however, beyond the focus of this article, since the resulting benefits relative to single-threaded programs are expected to be fairly independent of the choice of *r* versus *r*_*t*_ as a measure of internodal functional connectivity.

### Performance tests

In order to assess the performance of the programs described above, we compared them to three other programs: Matlab’s built-in function corrcoef, corr from Matlab’s Statistics Toolbox, and IPN_fastCorr, a function from the Matlab toolbox *ipnvoxelgraph* by X.N. Zuo. Experiments were conducted from within Matlab (R2011b) on a desktop computer with an Intel(R) Core(TM) i7-3960X CPU (3.30GHz) and 64GB main memory running Linux (Kernel 3.4). The C/MEX routines that are part of our programs were compiled using the GNU C compiler gcc (version 4.7.1, optimization level 3). In order to prevent programs from making use of multiple cores, Matlab was restricted to one CPU core.

Input data sets (**S**^*V*×*T*^) were generated using pseudo-random numbers of type float. While the length of time series *T* was fixed at *T*=200, the number of voxels *V* was varied between 10000 and 170000 in steps of 10000. The maximum number of voxels, 170000, follows from the fact that storing the resulting matrix (upper triangular part) requires 53.83 GB ($\frac{V(V-1)}{2}$floats, 4 bytes per float). Since the machine used has 64GB of main memory, this seemed a sensible choice in order to leave some memory for other applications and subsequent processing of the matrix. Because corrcoef, corr, and IPN_fastCorr return the complete symmetric matrix, they were only tested using input data sets with up to 120000 voxels corresponding to 53.64 GB of memory required to hold the matrix (*V*^2^floats).

## Results

### Correlation estimation

**Figure 3:**
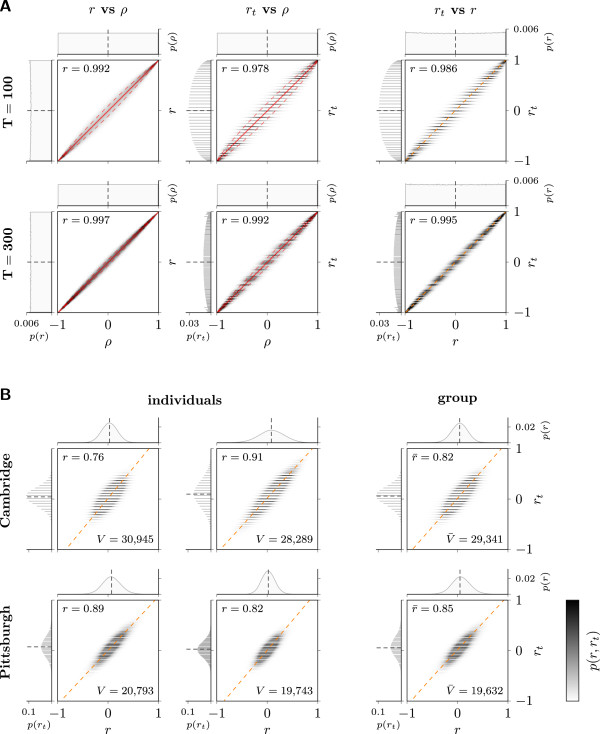
**Comparison of correlation estimates*r* and*r***_***t***_**.** Each composite plot consists of a joint histogram and the corresponding marginal histograms. Joint probabilities were mapped to grayscale intensities. A: Results for synthetic data. Top row: *T*=100. Bottom row: *T*=300. Left and mid column: *r* vs. *ρ* and *r*_*t*_ vs. *ρ*, respectively; mean (solid) and mean±STD (dashed) per *ρ*-bin shown in red. Right column: *r*_*t*_ vs. *r*; Deming regression line (dashed) shown in orange. B: Results for fMRI data (*r*_*t*_ vs. *r*). Top row: Cambridge data set. Bottom row: Pittsburgh data set. Left and mid column: Based on correlation matrices **C**_*r*_ and $C_{r_{t}}$ from two individual subjects. Right column: Based on *all* subject-specific correlation matrices **C**_*r*_ and $C_{r_{t}}$. Deming regression line (dashed) shown in orange.

Overall, the correlation estimation from dichotomized data using *r*_*t*_ yielded results strongly resembling those obtained through estimation from continuous data using *r*. For synthetic data, as expected, *r* and *r*_*t*_ exhibited linear relationships to *ρ* and also to each other (Figure 3A). Standard deviations (with respect to means per *ρ*-bin) were greater for *r*_*t*_ than for *r*, but were reasonably small for both. Peaking at *ρ*=0 with 0.101 and 0.058 (*r*), and 0.158 and 0.09 (*r*_*t*_), for *T*=100 and *T*=300, respectively, they exhibited a gradual decrease towards the range limits of *ρ*. Naturally, deviations from *ρ* were larger for *T*=100 than for *T*=300, because for *T*=100 each calculated correlation is based on fewer values than for *T*=300. Close inspection of the mean signed differences MSD(*r*_*t*_,*ρ*) and MSD(*r*,*ρ*) revealed a small systematic bias of both *r* and *r*_*t*_ as estimators of *ρ* (Additional file 1: Figure S3). The expected value of *r*, *E*(*r*), can be approximated by $E(r)=\rho -\rho (\frac{1-\rho^{2}}{2n})$[47,48]. *E*(*r*)−*ρ* closely matched the empirical results from the simulation represented by MSD(*r*,*ρ*), while MSD(*r*_*t*_,*ρ*) follows a curve of similar shape but larger amplitude. The Pearson correlation between *r* and *ρ*, *r*_*t*_ and *ρ*, and *r*_*t*_ and *r*, amounted to 0.992 (0.997), 0.978 (0.992), 0.986 (0.995), respectively, for T=100 (T=300).

For fMRI data, *r*_*t*_ and *r* followed a linear relationship in both data sets, although there was a slight counterclockwise rotation about the origin as reflected in a slope >1 as opposed to 1 for a perfect relation *r*_*t*_=*r* (Figure 3B). This suggests only limited deviation from the assumption of pairwise bivariate normality, and, moreover, indicates that *r*_*t*_-based graphs closely resemble *r*-based graphs. Furthermore, the results for the Cambridge data set (*T*=119) showed a greater variance than those for the Pittsburgh data set (*T*=275). Since this feature was also observed in the results for the synthetic data sets, we presume that this was caused by the smaller sample size for the calculation of each sample correlation. The overall correlation between *r* and *r*_*t*_ amounted to 0.82 (Cambridge) and 0.85 (Pittsburgh).

Note that the vertical gaps in bin occupation in those histograms involving *r*_*t*_ are due to the fact that *r*_*t*_ can attain a distinct set of values only, as explained earlier. The number of attainable values, and hence the (potential) performance of *r*_*t*_ as an estimator, increases with *T*.

### Node degree

**Figure 4:**
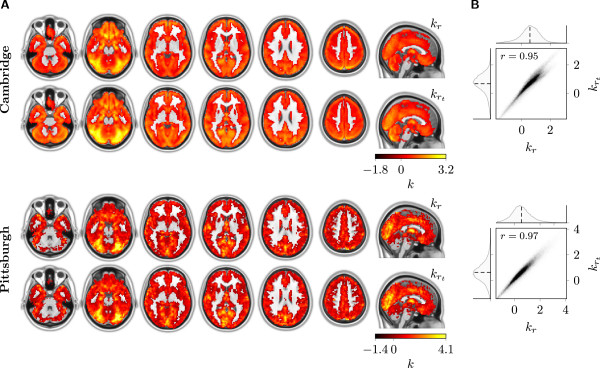
**Comparison of node degrees*k* from*r*- (*k***_***r***_**) and*r***_***t***_**-based (**$k_{r_{t}}$**) binary graphs.** Subject-specific graphs were derived from the correlation matrices **C**_*r*_ and $C_{r_{t}}$ using a density threshold of *κ*=0.01 corresponding to correlation thresholds of *r*=0.48±0.06 and *r*_*t*_=0.53±0.04 (Cambridge) and *r*=0.46±0.08 and *r*_*t*_=0.49±0.07 (Pittsburgh), respectively. Individual degree maps were spatially transformed to MNI space to derive group average maps. Note that the degrees were standardized for each subject before averaging, resulting in ranges that one would not commonly expect for degrees. For details see text. **A** Group average degrees on top of axial slices of the MNI brain (shown in neurological convention). Top row: *k*_*r*_. Bottom row: $k_{r_{t}}$. **B** Joint distribution of *k*_*r*_ and $k_{r_{t}}$. The composite plots consist of a joint histogram and the corresponding marginal histograms. Joint probabilities were mapped to grayscale intensities.

Group average node degree maps from *r*- and *r*_*t*_-based binary graphs **B**_*r*_ and $B_{r_{t}}$ (derived from **C**_*r*_ and $C_{r_{t}}$, respectively, using a density threshold of *κ*=0.01) are presented in Figure 4. Other thresholds (*κ*∈{0.05,0.1}) led to similar results and are hence not shown. In accordance with the strong correlation between *r* and *r*_*t*_ reported in the previous section, both approaches yielded highly similar spatially distributed node degree maps (Figure 4A). In line with this, *k*_*r*_ and $k_{r_{t}}$ were very strongly correlated ($r(k_{r},k_{r_{t}})=0.95$ for Cambridge and $r(k_{r},k_{r_{t}})=0.97$ for Pittsburgh; Figure 4B), although degrees tended to be slightly higher for *r*_*t*_-based than for *r*-based graphs. Prominent clusters of high-degree nodes were found within circumscribed regions of the occipital (cuneus, precuneus, fusiform and lingual gyri), parietal (intraparietal sulcus, superior parietal lobe, temporoparietal junction), temporal (superior temporal gyrus, temporal pole, amygdala) and frontal lobes (medial orbitofrontal and rostral ventromedial prefrontal cortex) with a similar distribution pattern as reported in previous work employing *r*-based node degree mapping [15,27,31].

### Performance tests

**Figure 5:**
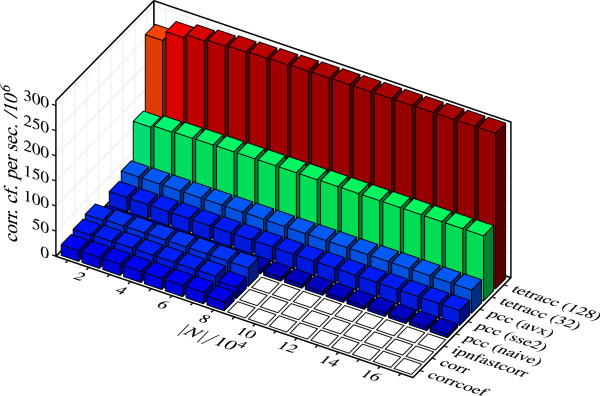
**Performance comparison for computation of correlation matrices.** Results are averages from 10 runs on a desktop computer with an Intel(R) Core(TM) i7-3960X CPU (3.3GHz) and 64GB main memory. All programs were restricted to one CPU core. Length of time series *T* was fixed at *T*=200. |*N*|: number of nodes. The programs corrcoef (Matlab built-in), corr (Matlab Statistics Toolbox), IPN_fastCorr (X.N. Zuo), and pcc (three variants) computed **C**_*r*_, while tetracc (two variants) computed $C_{r_{t}}$. The performance of each program is given as the number of correlation coefficients computed per second.

The most time-consuming step when constructing a graph from fMRI data consists in the computation of a functional connectivity matrix. We compared the performance of our programs on this task to three other programs: Matlab’s built-in function corrcoef, corr from Matlab’s Statistics Toolbox, and IPN_fastCorr, a function from the Matlab toolbox *ipnvoxelgraph* by X.N. Zuo. Table 1 shows memory requirements, execution times, and speedups relative to corrcoef which was selected as reference since it is available to any Matlab user out of the box and we therefore assume that it has a higher prevalence than corr or IPN_fastCorr. Figure 5 illustrates the performance in terms of data troughput measured in correlation coefficients per second. This measure does not depend on the performance of a reference program and offers more immediate access to the key results. In this sense, it is complementary to Table 1.

**Table 1:** Performance comparison for computation of correlation matrices

**|** ***N*** **|/10**^**3**^		corrcoef	corr		IPN_fastCorr			pcc/naive		pcc/SSE2		pcc/AVX		tetracc/32		tetracc/128	
	***m*** **[GB]**	***t*** **[s]**	***t*** **[s]**	***s*** **[*×*]**	***t*** **[s]**	***s*** **[*×*]**	***m*** **[GB]**	***t*** **[s]**	***s*** **[*×*]**	***t*** **[s]**	***s*** **[*×*]**	***t*** **[s]**	***s*** **[*×*]**	***t*** **[s]**	***s*** **[*×*]**	***t*** **[s]**	***s*** **[*×*]**
10	0.4	2.3	1.8	1.29	1.5	1.55	0.2	7.2	0.32	1.7	1.39	1.1	2.16	0.4	5.65	0.2	12.58
20	1.5	9.1	6.8	1.34	5.6	1.63	0.7	28.8	0.32	6.7	1.35	4.3	2.11	1.6	5.71	0.7	13.17
30	3.4	20.2	14.9	1.35	12.4	1.63	1.7	64.9	0.31	15.1	1.34	9.7	2.09	3.5	5.70	1.5	13.33
40	6.0	36.1	26.8	1.35	22.0	1.64	3.0	115.4	0.31	26.9	1.34	17.3	2.09	6.3	5.76	2.7	13.55
50	9.3	55.9	41.5	1.35	34.3	1.63	4.7	180.3	0.31	42.2	1.32	27.0	2.07	9.8	5.72	4.1	13.53
60	13.4	80.3	59.5	1.35	49.3	1.63	6.7	259.4	0.31	60.5	1.33	38.7	2.07	14.0	5.73	5.9	13.57
70	18.3	109.5	80.9	1.35	67.0	1.63	9.1	352.9	0.31	82.4	1.33	52.6	2.08	19.1	5.75	8.0	13.65
80	23.8	180.1	143.4	1.26	87.5	2.06	11.9	461.4	0.39	107.7	1.67	69.1	2.61	24.9	7.24	10.4	17.24
90	30.2						15.1	584.3		137.1		87.9		31.4		13.2	
100	37.3						18.6	721.0		168.7		107.8		38.7		16.3	
110	45.1						22.5	872.1		203.1		130.3		46.8		19.6	
120	53.6						26.8	1037.6		242.0		154.9		55.6		23.4	
130	63.0						31.5	1217.5		284.3		181.6		65.2		27.4	
140	73.0						36.5	1411.7		329.4		210.2		75.6		31.7	
150	83.8						41.9	1620.2		377.9		241.3		86.7		36.4	
160	95.4						47.7	1845.5		430.7		276.4		98.6		41.4	
170	107.7						53.8	2085.0		487.8		313.2		111.3		46.7	

Results are averages from 10 runs on a desktop computer with an Intel(R) Core(TM) i7-3960X CPU (3.3GHz) and 64GB main memory. All programs were restricted to one CPU core. Length of time series *T* was fixed at *T*=200. |*N*|: number of nodes. The programs corrcoef (Matlab built-in), corr (Matlab Statistics Toolbox), IPN_fastCorr (X.N. Zuo), and pcc (three variants) computed **C**_*r*_, while tetracc (two variants) computed $C_{r_{t}}$. *m* [GB]: memory requirements for result in GB; *t* [s]: elapsed time in seconds; *s* [ ×]: speedup relative to corrcoef.

In line with expectations, execution times increased quadratically with the number of nodes for all programs (Table 1). While pcc’s basic variant (pcc/naive) was considerably slower than corrcoef (speedup 0.31×), its SSE2- and AVX-based variants achieved speedups around 1.34× and 2.08×, respectively. The performance of corr (speedup 1.35×) was comparable to that of pcc/SSE2, while IPN_fastCorr (speedup 1.63×) ranked between pcc/SSE2 and pcc/AVX. The tetracc variants (32 and 128) were considerably faster than all programs computing *r* with speedups (relative to corrcoef) around 5.7× and 13.5×, respectively.

As an aside, we note that IPN_fastCorr as well as pcc and tetracc scaled better with the number of cores than corrcoef and corr. For example, using 6 cores and a data set of 50000 nodes (*T*=200), the speedups were 1.16× (corr), 2.46× (IPN_fastCorr), 2.32× (pcc/SSE2), 3.42× (pcc/AVX), 9.44× (tetracc/32), and 20.22× (tetracc/128), compared to corrcoef’s execution time of 18.5 seconds. Using the same data set but only 1 core the respective speedups were 1.35×, 1.63×, 1.33×, 2.08×, 5.7×, and 13.5× as compared to corrcoef’s execution time of 55.9 seconds (Table 1). Thus, the speedup gained by using 6 cores instead of 1 amounts to 4.56× (IPN_fastCorr), 5.29× (pcc/SSE2), 4.98× (pcc/AVX), 4.98× (tetracc/32), and 4.49× (tetracc/128), while corr and corrcoef gain only 2.6× and 3×, respectively.

Note that pcc and tetracc require only half the amount of memory (column 8) that corrcoef, corr, and IPN_fastCorr require (column 2), because they store only half of the symmetric matrix for memory efficiency (Table 1). In addition, corrcoef, corr, and IPN_fastCorr failed with an out-of-memory error for input data sets with 90000 or more nodes. We assume that these programs internally use more memory than we expected, since the resulting matrix of correlation coefficients would require less than half of the available memory (90000^2^·4Byte=30.17GB). Hence, the speedups of the remaining programs compared to corrcoef could not be computed for *T*≥90000.

## Discussion

Graph-based functional connectivity analysis at the level of individual voxels allows for spatially fine-grained characterization of functional networks in the human brain. However, with high-resolution data sets, such analyses can become infeasible due to the computational demands involved. Most previous studies investigating voxel-level functional connectivity graphs relied on Pearson’s *r* for estimating internodal functional connectivity [2,8,13,15,21-27,29-31]. As demonstrated here, the tetrachoric correlation coefficient *r*_*t*_ constitutes a time-efficient alternative to *r* as a measure of functional connectivity.

In order to reduce the computational costs associated with the analysis of voxel-level graphs, previous studies reduced the data’s spatial resolution [15,26,27], spatially restricted the graphical edges incorporated into the analysis [21], or utilized parallel computing [31]. In contrast, efficiency benefits from *r*_*t*_-based graph construction are achieved without sacrificing spatial resolution, disregarding graphical edges, or exploiting parallel computing. An open source software package containing the created programs is freely available for download [44]. Note that parallel versions of *r*- and *r*_*t*_-based graph construction have been implemented in addition to the sequential ones, thus providing additional efficiency gains that depend on the number of available processors. While this aspect is not the main focus of this article, as the resulting benefits (relative to sequential implementations) can be expected to be fairly independent of the choice of *r* versus *r*_*t*_ as a measure of internodal functional connectivity, the parallel implementations are still included in the software package [44].

Even though the dichotomization procedure (a prerequisite to the computation of *r*_*t*_) entails discarding information in the time domain, important characteristics of the original data appear to be preserved. In applications to artificially generated as well as real fMRI data the new technique proved capable of closely reproducing results obtained in conventional ways. More specifically, the usefulness of the *r*_*t*_-based approach was assessed by comparison with *r* in estimating the correlation parameter *ρ* of bivariate normal populations of known properties. In this setting, both the bias and standard deviation were greater for *r*_*t*_ than for *r*, but still reasonably small. Thus, *r*_*t*_-based correlation estimation yielded results closely resembling those obtained when using *r*. Beyond that, *r*- and *r*_*t*_-based graph construction and node degree computation were carried out for real fMRI data. A strong linear relationship was found between *r*- and *r*_*t*_-based correlations indicating that *r*_*t*_-based graphs closely resemble *r*-based graphs, since the graphs are derived from the correlation matrices. In line with this, the spatial distribution of node degrees was highly similar for *r*- and *r*_*t*_-based graphs and also in good correspondence with previous work [15,27,31].

As data mining approaches are currently gaining momentum in the neuroimaging community [36,37,49,50], the amount of publicly available experimental data is steadily growing. Consequently, development and implementation of efficient exploratory methods, such as the one presented here, are necessary in order to take full advantage of this wealth of data, especially with respect to connectivity analyses [51]. Fast construction and subsequent analysis of graphs may thus open new avenues for applications, including those within a clinical setting, where the voxel-level approach may be of particular importance. It is worth noting in this context that voxel-level graph construction can operate at the original data resolution, thus avoiding the reduction of the analysis’ spatial sensitivity [4,24]. For example, disease-related patterns, once identified, may serve as connectivity-based biomarkers that could aid, guide, or facilitate diagnostics and may increase prediction accuracy with respect to disease occurrence, recurrence, severity, or treatment outcome. Here, again, efficient methods are essential to facilitate assessment of individual patients within a narrow time frame [52]. If combined with efficient tools for subsequent analysis, the presented methods for fast graph construction may also be useful for online evaluation of functional connectivity in the context of real-time fMRI. This would allow for connectivity-based adaptation of experimental stimulation and interaction with the subject, for example, in task-based fMRI studies, or neurofeedback-based training. Taken together, we believe that there is a multitude of applications (be them experimental or clinical) that could benefit from the methods presented here, highlighting the growing importance of efficient tools for graph-based analysis of voxel-level connectivity.

### Limitations

As illustrated by the results, the accuracy of a correlation estimate naturally increases with the number of data points, i.e., the number of scans. Along the same lines, it has recently been shown that the reliability of functional homogeneity increases with scan duration [53]. For both correlation estimators, it is therefore recommended to avoid a low number of scans (caused, for example, by a short scan duration, or a long TR, or both). Since the deviation from the population correlation *ρ* is generally higher for *r*_*t*_ than for *r*, a low number of scans will affect *r*_*t*_ more severely than *r*.

The main focus of this work lies with the comparison of *r* and *r*_*t*_ as functional connectivity estimators. To reduce the impact of preprocessing on the data’s correlation structure prior to this comparison, we limited the preprocessing of the fMRI data to a minimum. The effect of additional preprocessing steps, or a different preprocessing pipeline altogether, on the robustness of the proposed methods should be subject of future research. Unpublished results from our group indicate, however, that the comparability of *r* and *r*_*t*_ remains essentially consistent.

## Conclusions

Voxel-level graphs allow for spatially fine-grained analyses of functional connectivity networks. In order to reduce the considerable computational demands involved, many previous studies reduced the spatial resolution of the data. Here, a new method for graph construction—exploiting time series dichotomization and tetrachoric correlation estimation—was devised, efficiently implemented, and compared to the conventional approach based on continuous-valued data and Pearson’s *r*. In applications to artificially generated as well as real fMRI data the new technique proved capable of producing highly similar results. Through efficient bit-based implementation adapted to the dichotomized data the novel method runs an order of magnitude faster while the original spatial resolution of the data is retained. Hence, its demonstrated performance, not only in producing consistent results, but in obtaining them substantially faster, makes the new approach a sensible and economical alternative to customary practice. An open source software package containing the created programs is freely available for download [44].

## Endnotes

^a^ Deming regression is a linear regression method that accounts for errors in both variables.

^b^ International Consortium for Brain Mapping.

^c^ SSE2 was introduced by Intel with the Pentium 4. It is also supported by AMD CPUs starting with the Athlon 64. AVX is supported starting with the Sandy Bridge (Intel) and Bulldozer (AMD) microarchitectures.

^d^POPCNT became available starting with the Nehalem (Intel) and Barcelona (AMD) microarchitectures.

## Competing interests

The authors declare that they have no competing interests.

## Authors’ contributions

KL conceived of the study and carried out the data analyses with conceptual input from MG, CMS, and CB. KL and CB created the software package and carried out the performance tests. KL drafted the manuscript. MG, CMS, RK, and CB edited the manuscript. All authors read and approved the final manuscript.

## Supplementary Material

Additional file 1 Supporting information. Click here for file
