# Supplementary material for: Fast construction of voxel-level functional connectivity graphs
Source: BMC Neurosci. 2014 Jun 19;15:78. doi: 10.1186/1471-2202-15-78 (PMC4081498; doi:10.1186/1471-2202-15-78)
Supplement: Additional file 1 — Supporting information. [file 1471-2202-15-78-S1.pdf]

S Supporting Information

S.1 Dichotomization of FMRI Data

We derive a binary representation of fMRI voxel time series by considering for any given value the deviation from its median. The resulting binary, i.e., dichotomous, time series can be exploited to speed up subsequent analyses, as processing of binary data can be efficiently implemented based on bits. Furthermore, through dichotomization the data become addressable by specialized methods that are not applicable to the data in their original continuous-valued form [Loewe et al., 2013].

FMRI data are series of periodically acquired 3D images of continuous-valued intensities. One such series is denoted by $\boldsymbol{i} = (i_1, i_2, \ldots, i_T)$, where T is the number of points in time at which the intensity images i_k, $k \in \{1, \ldots, T\}$, are recorded. The individual images are organized in a regular 3D voxel grid of size $X \times Y \times Z$. In order to simplify the processing, the voxel coordinates (x, y, z) are mapped (in an essentially arbitrary, but fixed fashion) to a linear index v with $1 \leq v \leq V = X \cdot Y \cdot Z$. In this way a data set can be represented by a data matrix $\mathbf{S}^{V \times T} = (s_{v,k})$. By $\boldsymbol{s}_v = s_{v,*} = (s_{v,1}, s_{v,2}, \ldots, s_{v,T})$, that is, the v-th row of $\mathbf{S}$, we denote the time series of voxel v.

We use a simple binary discretization, i.e., a dichotomization, in order to assign to each value in a time series either '1' or '0'. Formally, the dichotomized time series $\boldsymbol{d_v}$ of a voxel v is given by $\boldsymbol{d}_v = (d_{v,1}, d_{v,2}, \ldots, d_{v,T}) = (\mathrm{d}(s_{v,1}), \mathrm{d}(s_{v,2}), \ldots, \mathrm{d}(s_{v,T})) \in \{0,1\}^T$, induced by the function $\mathrm{d}(s_{v,k}) = \mathrm{H}(s_{v,k} - \tilde{\boldsymbol{s}}_v)$, $k \in \{1, \ldots, T\}$, where $\tilde{\boldsymbol{s}}_v$ denotes the median of the values in $\boldsymbol{s}_v$ and H is the Heaviside step function, defined as $\mathrm{H}(x) = 0$ if $x < 0$ and $\mathrm{H}(x) = 1$ otherwise. In other words, $d_{v,k}$ is equal to '1' if the signal intensity value $s_{v,k}$ amounts at least to the median of its corresponding time series $\boldsymbol{s}_v$. Analogous to the matrix $\mathbf{S}^{V \times T}$, the corresponding dichotomized matrix is denoted by $\mathbf{D}^{V \times T} = (d_{v,k})$, and $\boldsymbol{d}_v = \boldsymbol{d}_{v,*}$ denotes its v-th row.

S.2 Mathematical Derivation

Due to the dichotomization scheme used, the lower integration limits of

$$\begin{aligned} p_{11} &= \int_{\Phi^{-1}(p_{\bullet 0})}^{\infty} \int_{\Phi^{-1}(p_{0\bullet})}^{\infty} f(z_x, z_y) \mathrm{d}z_x \mathrm{d}z_y, \\ &= \frac{1}{2\pi\sqrt{1-r_t^2}} \int_{\Phi^{-1}(p_{\bullet 0})}^{\infty} \int_{\Phi^{-1}(p_{0\bullet})}^{\infty} \exp\left(-\frac{z_x^2 - 2r_t z_x z_y + z_y^2}{2(1-r_t^2)}\right) \mathrm{d}z_x \mathrm{d}z_y \end{aligned}$$

become zero, as $p_{\bullet 0} = p_{\bullet 1} = p_{0\bullet} = p_{1\bullet} = 0.5$ and thus $\Phi^{-1}(p_{\bullet 0}) = \Phi^{-1}(p_{0\bullet}) = \Phi^{-1}(0.5) = 0$. Transformation from cartesian to polar coordinates through the substitutions

$$\begin{aligned} z_x &= r\cos\varphi \\ z_y &= r\sin\varphi \\ z_x^2 + z_y^2 - 2r_t z_x z_y &= r^2(1 - 2r_t\cos\varphi\sin\varphi) = r^2(1 - r_t\sin(2\varphi)) \\ \mathrm{d}z_x\mathrm{d}z_y &= r\mathrm{d}r\mathrm{d}\varphi \end{aligned}$$

yields

$$\begin{aligned} p_{11} &= \frac{1}{2\pi\sqrt{1-r_t^2}} \int_0^{\frac{\pi}{2}} \int_0^{\infty} r\exp\left(-\frac{r^2(1 - r_t\sin(2\varphi))}{2(1-r_t^2)}\right) \mathrm{d}r\mathrm{d}\varphi \\ &= \frac{1}{2\pi\sqrt{1-r_t^2}} \int_0^{\frac{\pi}{2}} \left[\frac{-(1-r_t^2)}{1 - r_t\sin(2\varphi)} \exp\left(-\frac{r^2(1 - r_t\sin(2\varphi))}{2(1-r_t^2)}\right)\right]_0^{\infty} \mathrm{d}\varphi \\ &= \frac{\sqrt{1-r_t^2}}{2\pi} \int_0^{\frac{\pi}{2}} \frac{\mathrm{d}\varphi}{1 - r_t\sin(2\varphi)}. \end{aligned}$$

Using $\displaystyle\int \frac{\mathrm{d}\varphi}{1 - r_t\sin(2\varphi)} = \frac{1}{\sqrt{1-r_t^2}} \arctan\left(\frac{\tan\varphi - r_t}{\sqrt{1-r_t^2}}\right) + const.$

we proceed with

$$p_{11} = \frac{\sqrt{1-r_t^2}}{2\pi}\left[\frac{1}{\sqrt{1-r_t^2}}\arctan\left(\frac{\tan\varphi - r_t}{\sqrt{1-r_t^2}}\right)\right]_0^{\frac{\pi}{2}}$$
$$= \frac{\sqrt{1-r_t^2}}{2\pi}\left[\frac{\pi}{2\sqrt{1-r_t^2}} - \frac{1}{\sqrt{1-r_t^2}}\arctan\left(-\frac{r_t}{\sqrt{1-r_t^2}}\right)\right] = \frac{1}{4} + \frac{1}{2\pi}\arctan\left(\frac{r_t}{\sqrt{1-r_t^2}}\right)$$
$$= \frac{1}{2\pi}\arccos(-r_t). \quad (1)$$

By solving for r_t, we obtain

$$r_t = -\cos(2\pi p_{11}). \quad (2)$$

The same result also arises as a by-product of Pearson's holistic examination of the problem within the framework of series expansion [Pearson, 1900] (page 7, 20th equation). In contrast, the derivation we presented here focuses only on the special case at hand (where all marginal probabilities are equal) and may thus be more easily comprehensible.

S.3 Supplementary Figures

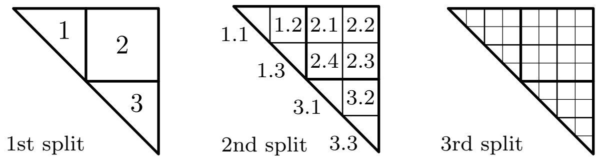

Figure S.1.
The first three splits into smaller and smaller blocks in the recursion of our cache-oblivious algorithm.

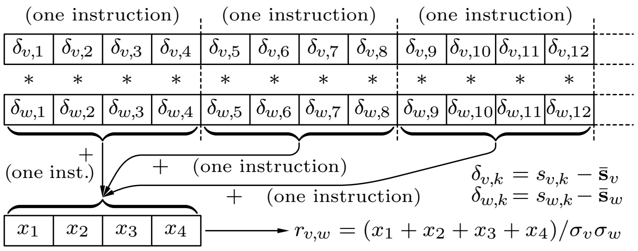

Figure S.2.
Exploiting SIMD instructions for the correlation computation (SSE2 or AVX, four sums in parallel).

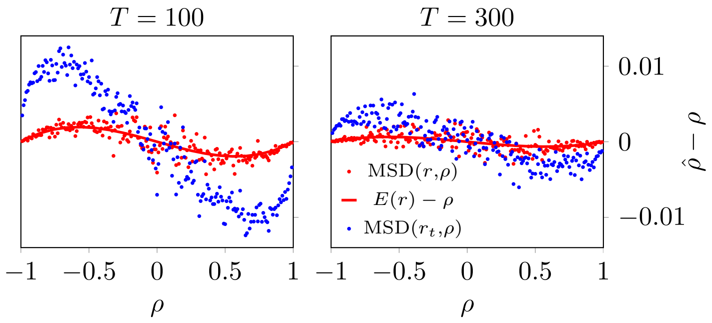

Figure S.3.
Both r and r_t are biased estimators of ρ. MSD(r,ρ) and MSD(r_t,ρ) for $T = 100$ and $T = 300$.

References

Loewe, K. et al. (2013). "Mining Local Connectivity Patterns in fMRI Data." In: *Towards Advanced Data Analysis by Combining Soft Computing and Statistics*. Ed. by C. Borgelt et al. Vol. 285. Studies in Fuzziness and Soft Computing. Springer Berlin Heidelberg, pp. 305–317.

Pearson, K. (1900). "Mathematical Contributions to the Theory of Evolution. VII. On the Correlation of Characters not Quantitatively Measurable". In: *Philosophical Transactions of the Royal Society of London* 195, pp. 1–47.
